# Ginsenoside Rg1 Ameliorates Behavioral Abnormalities and Modulates the Hippocampal Proteomic Change in Triple Transgenic Mice of Alzheimer's Disease

**DOI:** 10.1155/2017/6473506

**Published:** 2017-10-24

**Authors:** Lulin Nie, Junxia Xia, Honglian Li, Zaijun Zhang, Ying Yang, Xinfeng Huang, Zhendan He, Jianjun Liu, Xifei Yang

**Affiliations:** ^1^Key Laboratory of Modern Toxicology of Shenzhen, Shenzhen Center for Disease Control and Prevention, Shenzhen 518055, China; ^2^Department of Obstetrics, Shenzhen People's Hospital, The Second Clinical Medical College of Jinan University, Shenzhen 518020, China; ^3^Department of Histology and Embryology, Tongji Medical College, Huazhong University of Science and Technology, Wuhan 430030, China; ^4^Institute of New Drug Research and Guangzhou Key Laboratory of Innovative Chemical Drug Research in Cardio-cerebrovascular Diseases, Jinan University College of Pharmacy, Guangzhou 510632, China; ^5^Department of Pathophysiology, Tongji Medical College, Huazhong University of Science and Technology, Wuhan 430070, China; ^6^School of Pharmacy, Health Science Center, Shenzhen University, Shenzhen 518055, China

## Abstract

Alzheimer's disease (AD) is one of the most common neurodegenerative diseases, so far, there are no effective measures to prevent and cure this deadly condition. Ginsenoside Rg1 (Rg1) was shown to improve behavioral abnormalities in AD; however, the potential mechanisms remain unclear. In this study, we pretreated 7-month-old 3xTg-AD mice for 6 weeks with Rg1 and evaluated the effects of Rg1 on the behaviors and the protein expression of hippocampal tissues. The behavioral tests showed that Rg1 could improve the memory impairment and ameliorate the depression-like behaviors of 3xTg-AD mice. Proteomic results revealed a total of 28 differentially expressed hippocampal proteins between Rg1-treated and nontreated 3xTg-AD mice. Among these proteins, complexin-2 (CPLX2), synapsin-2 (SYN2), and synaptosomal-associated protein 25 (SNP25) were significantly downregulated in the hippocampus of 3xTg-AD mice compared with the WT mice, and the treatment of Rg1 modulated the expression of CPLX2 and SNP25 in the hippocampus of 3xTg-AD mice. The expression of CPLX2, SYN2, and SNP25 was further validated by Western blot analysis. Taken together, we concluded that Rg1 could be a potential candidate drug to improve the behavioral deficits in AD via modulating the expression of the proteins (i.e., CPLX2, SYN2, and SNP25).

## 1. Introduction

Alzheimer's disease (AD) is an irreversible degenerative disease with brain dysfunction occurring among aged people, which is the main cause of dementia and affects 60%–65% of the world population [1, 2]. According to the World Alzheimer Report 2016, the number of the patients with dementia is rapidly increasing due to aging. The report showed that there were 46.8 million people worldwide living with dementia in 2015 and this number will reach 131.5 million in 2050 [3]. Therefore, urgent effective treatment strategies against AD are desired.

The distinctive histopathological hallmarks of AD are extracellular senile plaques consisted of *β*-amyloid (A*β*) peptides, intracellular neurofibrillary tangles, neuronal loss, and synapse injury in some vulnerable regions such as the hippocampus [4–6]. In addition to cognitive impairment, neuropsychiatric symptoms such as depression-like behavior were prevalent in AD cases [7–10]. Although great advances have been achieved in the etiology of AD, the mechanisms of neurodegenerative disease remain largely unclear.

Ginsenoside Rg1, a pharmacological active component purified from the natural product ginseng, could cross the blood-brain barrier (BBB) and had a positive effect on the brain [11]. Several studies revealed the neuroprotective properties of Rg1 in neurodegenerative diseases such as AD [12, 13]. Rg1 could ameliorate cognitive impairment in the mouse model of AD, improve the learning and memory abilities [14, 15], decrease the levels of cerebral A*β* [16], maintain hippocampal neuron activity [17], and prevent cellular apoptosis induced by A*β* accumulation [18]. On the other hand, Rg1 also could protect against brain aging by enhancing the scavenging of free radicals in the brain [19]. However, the mechanisms underlying the protective effects of Rg1 against the behavioral abnormality and pathological changes are poorly understood.

In this study, we explored the effects of Rg1 treatment on memory and depression-like behaviors of 3xTg-AD mice and hippocampal proteome. It was worth mentioning that 3xTg-AD mice displayed obvious memory impairment and anxiety and depression-like behavior [20, 21]. By using two-dimensional fluorescence differential gel electrophoresis (2D-DIGE) with mass spectrometry, we identified the differentially expressed proteins in the hippocampus of triple transgenic mice of AD (3xTg-AD) with or without administration of Rg1 and revealed the potential key molecules that may be involved in memory deficit and depression-like behaviors in AD.

## 2. Material and Methods

### 2.1. Materials

Ginsenoside Rg1 was provided by Prof. Zhendan He, College of Life Sciences, Shenzhen University. The ginsenoside Rg1 was dissolved in saline and administered by intraperitoneal injection.

### 2.2. Animals and Treatment

The mice were purchased from Jackson Laboratory. In this study, we selected 7-month-old female 3xTg-AD mice harboring PS1_M146V_, APP_Swe_, and Tau_P301L_ transgenes (strain: B6; 129-Psen1^tm1Mpm^ Tg [APPSwe, tauP301L] 1Lfa/Mmjax) and WT (strain: B6129SF2/J) mice. The 7-month 3xTg-AD mice were treated with Rg1 (20 mg/kg body weight) by intraperitoneal injection for 6 weeks [22]. In parallel, nontreated 3xTg-AD mice and WT mice were injected with saline. All animal experiments and procedures were approved by Shenzhen Center for Disease Control and Prevention. All efforts were made to minimize animal suffering and reduce the number of mice used.

### 2.3. Behavioral Evaluation

All the behavior tests were conducted in a quiet room, the experimental mice were transferred to the behavioral room 2 h before the assessment. In this study, the anxiety behavior of mice was tested by the open field test and the elevated plus maze test, the depression behavior of mice was tested by the tail suspension test, and the learning and memory ability of mice was measured by the Morris water maze test. The interval between tests was two days.

#### 2.3.1. Open Field Test

The open field test was performed as previously described by Prut and Belzung [23]. The apparatus consisted of a brightly illuminated (120 lux) square arena of about 0.5 m width closed by a wall 0.45 m high. The whole arena was divided into 16 square areas (12.5 cm × 12.5 cm). “Center” was consisted of four small squares of the center, and “corner” was made up of other squares. The camera was installed above the center of the device. Experimental mice were placed in the “center” and allowed to move freely in 5 min. The degree of anxiety was assessed based on the activity time and distance of the mice in the center. The apparatus was cleaned with 75% ethanol solution between tests.

#### 2.3.2. Elevated Plus Maze Test

The elevated plus maze test was performed as previously described by Belzung and Griebel [24]. It consisted of a cross of two closed arms (50 × 10 cm) and two open arms (50 × 10 cm). The mice were placed in the center and allowed to explore the maze for 5 min. The lesser time and distance of the animals entering into open arms suggested that they were more anxious.

#### 2.3.3. Tail Suspension Test

The tail suspension test was performed as previously described by Steru et al. [25]. Each mouse was suspended 50 cm above the floor for 5 min by sticking its tail. The mice were considered immobile only when they were fully motionless. In this study, the degree of depression was measured by the immobility ratio of mice.

#### 2.3.4. Morris Water Maze Test

Morris water maze was performed as previously described by Deng-Bryant et al. [26]. It was used to evaluate spatial learning and memory of the mice. The water maze consisted of a circular pool (1.7 m in diameter) filled with milk (0.3 m in depth, 22°C) and a circular white escape platform (0.1 m in diameter) submerged 2 cm below the surface of the water. The water maze was subdivided into four quadrants (I–IV), the four quadrants were marked with triangular, circular, cross, and square, respectively. All the mice were trained for 5 consecutive days (4 trials /day) and the platform was in a fixed position. In the training stage, each mouse was given 60 s time to explore the platform, if the mice cannot find the platform within 60 s, the experimenter led the mice to stay on the platform 15 s. The latency of each mice was recorded. After 1 week, the mice were detected for 120 s without the platform in the pool. The swimming path was recorded by using the video tracking system.

### 2.4. Sample Preparation

The experimental mice were sacrificed after the behavioral tests. The bilateral hippocampus of mice was dissected on ice, and all samples were stored in −80°C. Samples were ultrasounded (3 s on, 5 s off for 3 min) in lysis buffer (7 M urea, 2), then the samples were centrifuged at 4°C, 12000*g* for 1 h. The protein solution discarded salts and impurities by ultrafiltration. The protein concentration was measured with 2-D Quant Kit (GE Healthcare, USA). The samples (pH = 8.5) were diluted to 5 *μ*g/*μ*L according to the protein quantification. All samples were stored in −80°C.

### 2.5. DIGE Labeling of Protein Samples

The DIGE labeling of protein samples was performed as previously by Iadevaia et al. [27]. Each CyDye dye was dissolved in 99.8% anhydrous N,N-dimethylformamide (DMF, Sigma 227056) to a final dye concentration of 1 nmol/*μ*L as stock solution. The stock solution was diluted with DMF to 200 pmol/*μ*L of working solution. The protein of differential group was treated with 200 pmol Cy3 (GE Healthcare, 25-8008-61) or Cy5 (GE Healthcare, 25-8008-62) dye markers for comparison on the same gel, and the internal standard was labeled with Cy2 (GE Healthcare, 25-8008-62). In brief, 25 *μ*g of each protein sample was labeled with 200 pmol CyDyes on ice in the dark for 30 min and then quenched with 10 mmol lysine (Sigma, L5626) for 10 min. Then, the Cy5-, Cy3-, and Cy2-labeled samples were mixed together, and each mixture was added with an equal volume of 2× lysis buffer (8 M urea, 2% CHAPS, 0.2% DTT, 2% (*v*/*v*) IPG buffer, pH 3–11 nonlinear, 0.002% bromophenol blue) and incubated on ice for 10 min. The total volume of the sample was diluted to 450 *μ*L by adding hydration buffer.

### 2.6. Isoelectric Focusing and SDS-PAGE

The first-dimension isoelectric focusing (IEF) was performed using the Ettan IPGphor IEF system (GE Healthcare, USA). A total of 75 *μ*g of each labeled samples was applied on a 24 cm pH 3–11 NL Immobilized DryStrip (GE Healthcare). IEF was performed using the following conditions: 18 h at 50 V, 12 h at 300 V, 2 h at 500 V, 2 h at 1000 V, and 8 h at 8000 V. Each time, four or six IPG strips were run in parallel. After the first-dimension IEF, each strip was equilibrated for 15 min in the reducing equilibration buffer (6 mol/L urea, 30% (*v*/*v*) glycerol, 75 mmol/L Tris-HCl buffer (pH 8.8), 2% (*w*/*v*) SDS, and 1% (*w*/*v*) DTT) at room temperature. Subsequently, each strip was re-equilibrated in the similar buffer containing 4.5% iodoacetamide (IAA) instead of DTT for 15 min. After the equilibration, each equilibrated strip was loaded on the top of 12.5% SDS-PAGE gels (25 mM Tris, 192 mM glycine, 0.1% SDS, 0.02% bromophenol blue, 0.5% (*w*/*v*) agarose) for the second dimension. Gels were run in Ettan DALTsix Electrophoresis System (GE Healthcare), and the electrophoresis was conducted under the following conditions: 1 W/gel for 1 h and 10 W/gel for 5 h in the dark at 12°C. Gels were immediately scanned using a Typhoon Trio Variable Mode Imager (GE Healthcare).

### 2.7. Image Analysis

Gel images were visualized by the Image Quant TL software. Differential proteins were analyzed using DeCyder TM 2D software (version 6.5 GE Healthcare) Differential In-gel Analysis (DIA) and Biological Variance Analysis (BVA) modules. The fluorescent intensity of each protein spot was determined in the Cy3 or Cy5 channels and then normalized according to the corresponding fluorescent intensity of Cy2 spot. The normalized volume of a spot was compared across the gels between the replicate groups. The differential protein spots with significant difference (*p* < 0.05) were further analyzed.

### 2.8. In-Gel Digestion

A total of 1200 *μ*g of hippocampal protein was used to run 2-DE using the identical conditions as above. The gel was stained with Coomassie Brilliant Blue solution for 12 h, then the gel was washed many times until the protein spots were visible. The differential protein spots with significant difference (*p* < 0.05) were manually excised from Coomassie blue staining gel. Subsequently, the protein spots were washed with deionized water and destained with 25 mmol/L NaHCO_3_ solution (50% ACN, 50% H_2_O) for 1 h at 37°C. The spots were swollen and digested in a trypsin buffer (Promega Corp., WI, USA) for 12 h at 37°C. Then, peptides were analyzed by MALDI-TOF-MS/MS.

### 2.9. Mass Spectrometry and Database Searching

Protein identification was analyzed by MALDI-TOF-MS/MS (AB SCIEX MALDI-TOF/TOF 5800 mass spectrometer). For the MADL-TOF-MS, 0.6 *μ*L of each sample peptide extraction was spotted on a stainless steel target and dried at room temperature, then each spot was crystallized with 1 *μ*L 50% acetonitrile (ACN) and 0.1% trifluoroacetic acid (TFA) containing 10 mg/mL *α*-cyano-4-hydroxycinnamic acid (CHCA) and dried at room temperature. After the spectra were manually calibrated, database searching was carried out by MASCOT based on the SwissProt databases in *Mus musculus*. The search was performed with a tolerance on mass measurement of 100 ppm in MS mode and 0.5 Da in MS/MS mode. Protein pI and MW information were recorded to evaluate the identification of protein spots.

### 2.10. Gene Annotation

The Panther Bioinformatics Resource was used to carry out gene ontology annotation enrichment analysis. Panther analysis enabled the enrichment of biological process, molecular function, cellular components, and protein class (http://www.pantherdb.org/).

### 2.11. Western Blot Analysis

Hippocampal proteins from 3xTg-AD mice, 3xTg-AD-Rg1 mice, and WT mice were ultrasounded in 400 *μ*L lysis buffer (Beyotime, China) and 4 *μ*L protease and phosphatase inhibitor cocktail (Thermo Scientific, USA) on the ice, and then, the protein samples were centrifuged and collected. The protein concentration was measured with BCA protein assay kit (Thermo Scientific, USA). Each protein sample was added with loading buffer and heat for 7 min at 100. Protein samples were separated on 10% PAGE gels with 5% stacking gels and transferred to PVDF membranes. The membrane was soaked in TBST buffer containing 5% milk at room temperature for 2 hours. Subsequently, membranes were incubated with anti-SYN2 (rabbit polyclonal antibody, 1 : 3000), anti-SNP25 (rabbit polyclonal antibody, 1 : 3000), anti-complex 2 (rabbit polyclonal antibody, 1 : 3000), anti-PSD-95 (rabbit monoclonal antibody, 1 : 3000), and anti-*β*-actin (mouse monoclonal antibody, 1 : 3000) in TBST buffer for 1.5 h at room temperature. After washing with TBST three times, the membrane was incubated with homologous secondary antibody (anti-rabbit or anti-mouse IgG HRPs) in TBST buffer for 60 min. After repeatedly washing with TBST buffer, the membranes were developed using chemiluminescence reagents from an ECL kit (Pierce) and detected on a phosphorimager. The image of the membranes was analyzed by ImageQuant 1D software.

### 2.12. Statistical Analysis

Data were expressed as the mean ± SD and analyzed by using SPSS 19.0 statistical software (SPSS Inc., Chicago, Illinois, USA). The different means among the groups were evaluated by one-way ANOVA. For all the one-way ANOVA, post hoc tests were performed using LSD test. The level of significance was set at *p* < 0.05.

## 3. Results

### 3.1. Anti-Anxiety-Like Effects of Rg1 in 3xTg-AD Mice

**Figure 1:**
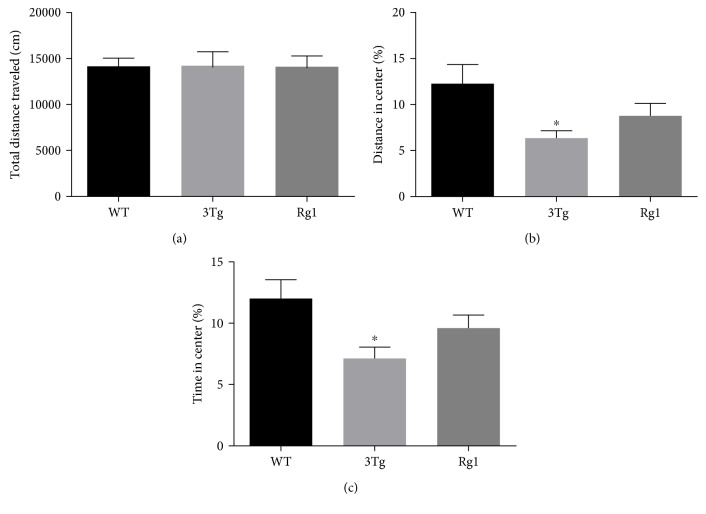
Anxiety-like behavior was measured by open field test. (a) Total distance traveled. (b) Distance traveled in the center of the open field. (c) Time spent in the center of the open field. The data were presented as mean ± SEM. ^∗^*p* < 0.05 versus WT mice. *n* = 10–15 for each group.

**Figure 2:**
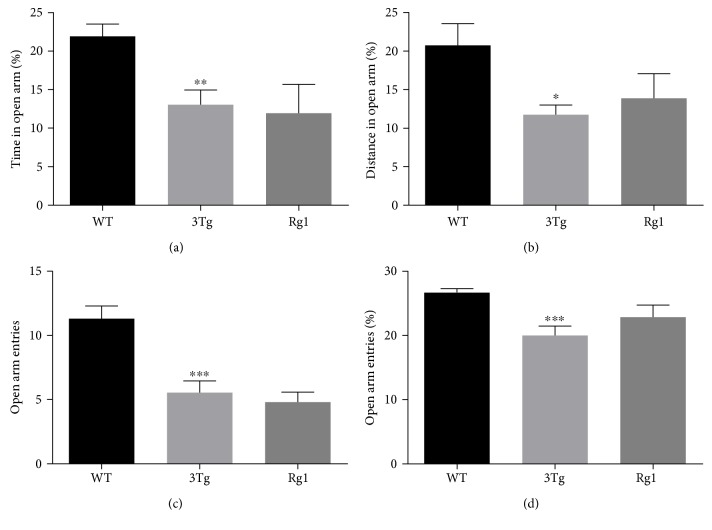
Anxiety-like behavior was measured by elevated plus maze test. (a) The percentage of the time spent in the open arms. (b) The percentage of the distance traveled in the open arms. (c) The number of entries into open arms. (d) The number of entries into open arms/number of entries to the open plus closed arms. The data were presented as mean ± SEM. ^∗^*p* < 0.05, ^∗∗^*p* < 0.01, and ^∗∗∗^*p* < 0.001 versus WT mice. *n* = 10–15 for each group.

The anti-anxiety-like activity of Rg1 was assessed by the open field test and the elevated plus maze test. The open field test data were summarized in Figure 1. The data showed no significant differences in the total distance traveled among the three groups (Figure 1(a)), while the nontreated 3xTg-AD mice showed significantly decreased percentage of time (*p* < 0.05) and distance (*p* < 0.05) in the center compared with the WT mice (Figures 1(b) and 1(c)). Rg1-treated 3xTg-AD mice showed an upward trend of the time spent and the distance traveled in the center compared with the nontreated 3xTg-AD mice (Figures 1(b) and 1(c)). The elevated plus maze test data are summarized in Figure 2. Compared with the WT mice, the percentage of time spent (*p* < 0.01; Figure 2(a)) and the percentage of the distance traveled (*p* < 0.05; Figure 2(b)) in open arms, the number of entries into the open arms (*p* < 0.001; Figure 2(c)), and the percentage of open arm entries (*p* < 0.001; Figure 2(d)) also were significantly decreased in the nontreated 3xTg-AD mice. Increased trend of both the percentage of distance (Figure 2(b)) and the entry frequency (Figure 2(d)) in open arms was observed after the treatment with Rg1 in 3xTg-AD mice. No differences were observed in the percentage of time in open arms (Figure 2(a)) and the number of entries into the open arms (Figure 2(c)) after the treatment with Rg1 in 3xTg-AD mice. These data suggested a mildly protective effect of Rg1 treatment against the anxiety-like behavior of 3xTg-AD mice.

### 3.2. Anti-Depression-Like Effects of Rg1 in 3xTg-AD Mice

**Figure 3:**
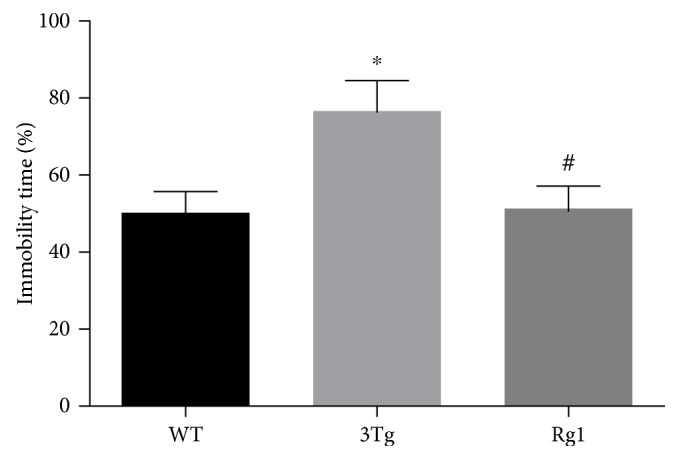
Depression-like behavior was measured by tail suspension test. The percentage of immobility time. The data were presented as mean ± SEM. ^∗^*p* < 0.05 versus WT mice; ^#^*p* < 0.05 versus 3xTg-AD mice. *n* = 10–15 for each group.

The depression-like behaviors of the mice were assessed by the forced swimming test, as depicted in Figure 3. Compared with the WT mice, the 3xTg-AD mice had a longer duration of immobility (*p* < 0.05), while the duration of immobility was significantly decreased after the treatment of Rg1 (*p* < 0.05). These data indicated that Rg1 could significantly alleviate depression-like behavior of 3xTg-AD mice.

### 3.3. Improved Spatial Learning and Memory of 3xTg-AD Mice by Rg1 Treatment

**Figure 4:**
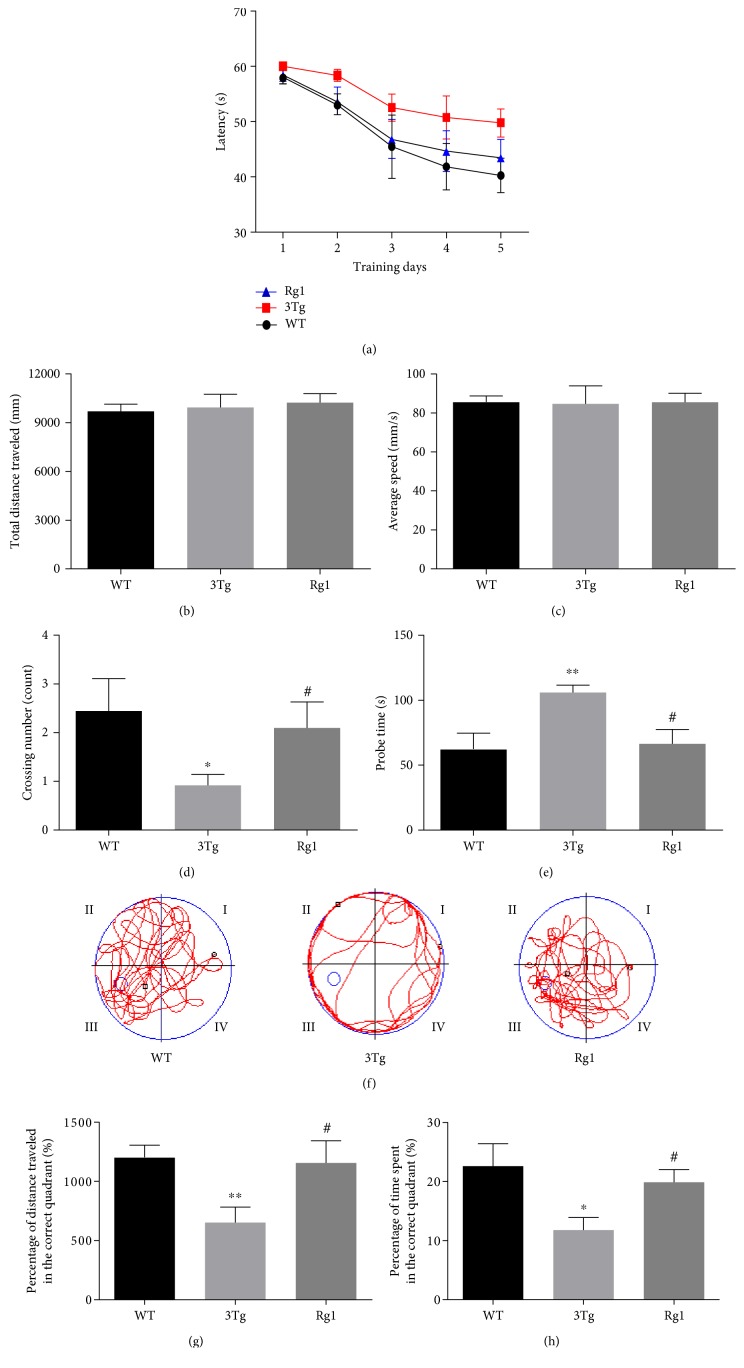
Memory behavior was measured by the Morris water maze. (a) Latency (s); (b) total distance traveled; (c) average speed; (d) crossing number; (e) probe time; (f) track diagram. (g) The percentage of distance traveled in the correct quadrant. (h) The percentage of time spent in the correct quadrant. The data were presented as mean ± SEM. ^∗^*p* < 0.05 and ^∗∗^*p* < 0.01 versus WT mice; ^#^*p* < 0.05 versus 3xTg-AD mice. *n* = 10–15 for each group.

The learning and memory ability of mice was measured by the Morris water maze test, as depicted in Figure 4. The total distance traveled (Figure 4(b)) and the average speed (Figure 4(c)) were not significantly different among the three groups, suggesting that the motor function of the experimental mice was not impaired. In the training period, the 3xTg-AD mice spent longer time reaching the platform on the last four days compared to the WT mice (Figure 4(a)). Rg1-treated 3xTg-AD mice displayed reduced escape latency compared to the 3xTg-AD mice (Figure 4(a)). These data indicated that Rg1 treatment improved the learning ability of AD transgenic mice. In the probe period, the platform crossing number of 3xTg-AD mice was significantly lower than the WT mice (*p* < 0.05; Figure 4(d)), while the probe time of 3xTg-AD mice was significantly higher than the WT mice (*p* < 0.01; Figure 4(e)). The percentage of distance traveled (*p* < 0.01; Figure 4(g)) and the percentage of time spent (*p* < 0.05; Figure 4(h)) in the target quadrant of 3xTg-AD mice were significantly decreased compared to the WT mice. Compared with the nontreated 3xTg-AD mice, the crossing number (*p* < 0.05; Figure 4(d)), the percentage of distance traveled (*p* < 0.05; Figure 4(g)), and the percentage of time spent (*p* < 0.05; Figure 4(h)) in the target quadrant were significantly increased, and the probe time was significantly decreased in 3xTg-AD mice after the treatment of Rg1. The representative movement tracks of three groups were shown in Figure 4(f). These data demonstrated that Rg1 treatment could improve the spatial learning and memory ability of 3xTg-AD mice.

### 3.4. Differentially Expressed Hippocampal Proteins among the WT Mice, Nontreated 3xTg-AD Mice, and Rg1-Treated 3xTg-AD Mice

**Figure 5:**
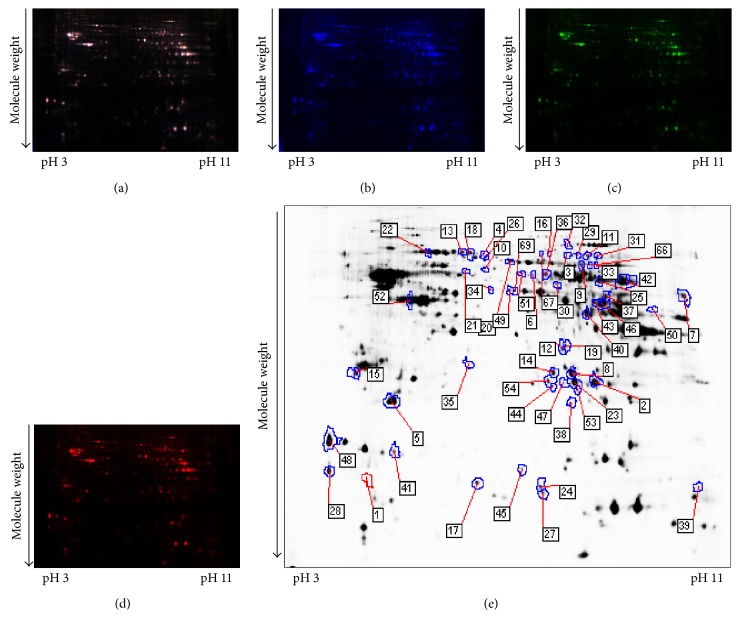
A representative 2D-DIGE gel image of hippocampal proteins from the WT mice and nontreated 3xTg-AD mice. Hippocampal proteins from the WT mice and nontreated 3xTg-AD mice were labeled with Cy3 or Cy5 dye, respectively (*n* = 6 for each group). An internal standard protein sample (a mixture of all hippocampus samples) was labeled with the Cy2 dye. The CyDye-labeled samples were combined, and the proteins were coseparated in the first dimension via IEF in 24 cm pH 3–11 nonlinear IPG strips, followed by separation in the second dimension via SDS-PAGE. Spots of interest were manually excised, digested, and subjected to identification by MALDI-TOF-MS/MS. (a) Cy2-labeled proteins as internal standards. (b) Cy3-labeled hippocampus proteins of WT mice. (c) Cy5-labeled hippocampus proteins of nontreated 3xTg-AD mice. (d) The merged image showing Cy2-, Cy3-, and Cy5-labeled proteins. (e) Greyscale 2D-DIGE gel image showing 47 differentially expressed protein spots identified by MALDI-TOF-MS/MS (black numbers with white square) in the hippocampus of nontreated 3xTg-AD mice compared with WT mice.

**Figure 6:**
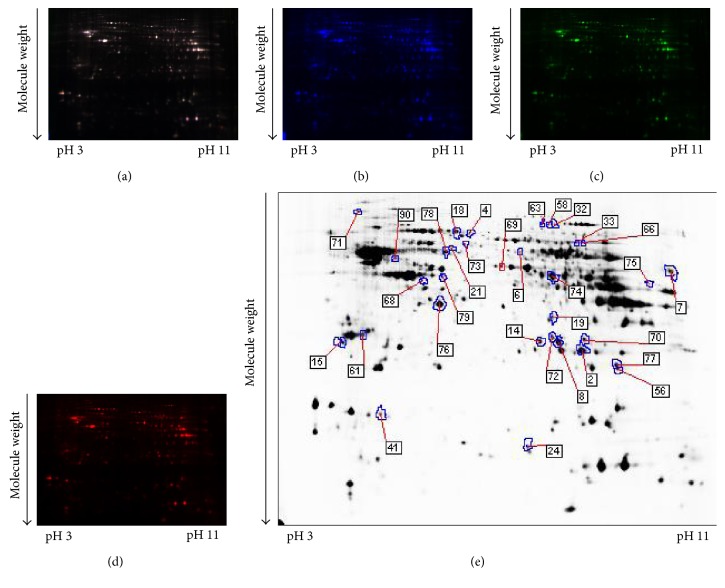
A representative 2D-DIGE gel image of hippocampal proteins from 3xTg-AD mice with or without Rg1 treatment. Hippocampal proteins from nontreated 3xTg-AD mice and Rg1-treated 3xTg-AD mice were labeled with Cy3 or Cy5 dye, respectively (*n* = 6 for each group). An internal standard protein sample (a mixture of all hippocampus samples) was labeled with the Cy2 dye. The CyDye-labeled samples were combined, and the proteins were coseparated in the first dimension via IEF in 24 cm pH 3–11 nonlinear IPG strips, followed by separation in the second dimension via SDS-PAGE. Spots of interest were manually excised, digested, and subjected to identification by MALDI-TOF-MS/MS. (a) Cy2-labeled proteins as internal standards. (b) Cy3-labeled hippocampus proteins of nontreated 3xTg-AD mice. (c) Cy5-labeled hippocampus proteins of melatonin-treated 3xTg-AD mice. (d) The merged image showing Cy2-, Cy3-, and Cy5-labeled proteins. (e) Greyscale 2D-DIGE gel image showing 28 differentially expressed protein spots identified by MALDI-TOF-MS/MS (black numbers with white square) in the hippocampus of Rg1-treated 3xTg-AD mice compared with nontreated 3xTg-AD mice.

To identify the potential molecules involved in the neuroprotection of Rg1, comparative proteomic analysis was performed on the hippocampal samples from the WT mice and 3xTg-AD mice with or without Rg1 treatment. Representative 2D-DIGE gel images of hippocampal proteins isolated from the three groups of mice were shown in Figures 5(a), 5(b), 5(c), and 5(d) and Figures 6(a), 6(b), 6(c), and 6(d). Spots with a fold change of 1.2 or greater and a *p* value ≤ 0.05 were recommended as differentially expressed protein spots. A total of 75 differentially expressed protein spots were annotated in the form of protein ID and identified with MS (Figures 5(e) and 6(e)). The mascot scores, protein names, theoretical molecular weight, *p* values, and fold-change levels of these differentially expressed proteins were displayed in Tables 1 and 2.

### 3.5. Identification of Differentially Expressed Hippocampal Proteins between WT and 3xTg-AD Mice

As depicted in Table 1, a total of 47 differentially expressed proteins were identified with MS between 3xTg-AD mice and the WT mice. These differentially expressed proteins were highly correlated with synaptic transmission, glycolytic process, response to stress, and negative regulation of apoptotic process. 10.6% of the differentially expressed proteins was related to synaptic transmission and 14.9% of the proteins was involved in glycolytic process. 17% of the proteins was involved in response to stress, and 6.4% of the proteins participated in the regulation of apoptotic process. Among these proteins, a total of 27 proteins, including memory-related proteins such as synaptosomal-associated protein 25 (SNP25), synapsin-2 (SYN2), and complexin-2 (CPLX2), in 3xTg-AD mice compared with WT mice were significantly downregulated, while a total of 20 proteins were significantly upregulated.

### 3.6. The Effect of Rg1 Treatment on the Protein Expression in Hippocampus of 3xTg-AD Mice

As depicted in Table 2, a total of 28 differentially expressed proteins were identified with MS between Rg1-treated 3xTg-AD mice and nontreated 3xTg-AD mice. Among these differentially expressed proteins, 14.3% of the proteins was related to synaptic transmission and 10.7% of the proteins was involved in glycolytic process. 21.4% of the proteins was involved in response to stress, and 10.7% of the proteins participated in the regulation of apoptotic process. A total of 16 proteins were downregulated in 3xTg-AD mice after the treatment of Rg1, and a total of 12 proteins were upregulated. Furthermore, we found that Rg1 treatment was able to modulate the expression of multiple synaptic proteins such as SNP25 and CPLX2.

### 3.7. Enrichment Analysis of the Differentially Expressed Protein

**Figure 7:**
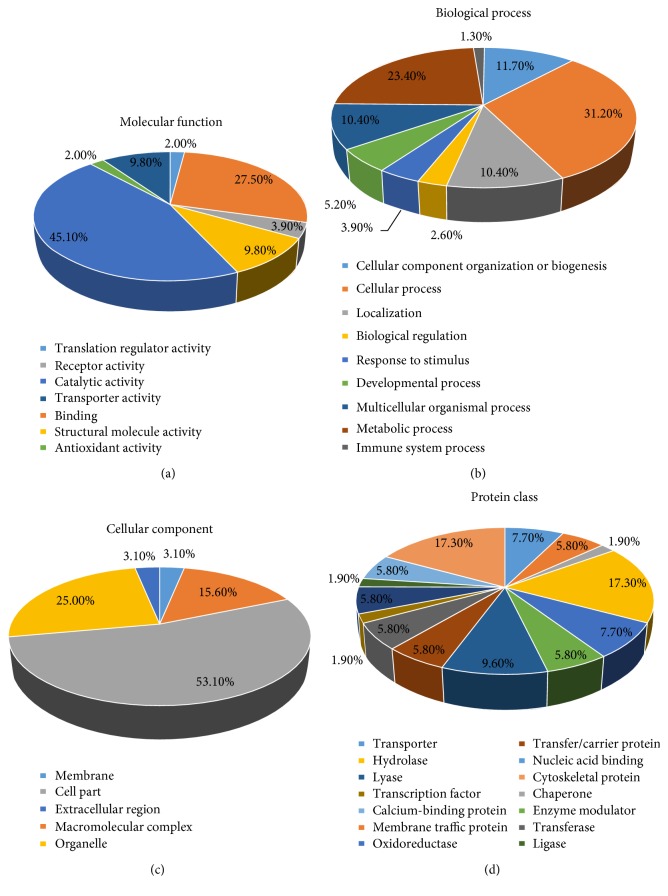
PANTHER functional enrichment analysis of differentially expressed proteins. (a) Enrichment analysis by molecular function. (b) Enrichment analysis by biological process. (c) Enrichment analysis by cellular component. (d) Enrichment analysis by protein class.

To investigate the biological function of the identified hippocampal proteins, we carried out Gene Ontology (GO) annotation search using the *Mus musculus* databases (http://www.pantherdb.org/). GO annotations were available for the 75 identified proteins included repeats, and the 61 proteins were grouped according to their molecular function, biological processes, cellular component, and protein class (Figures 7(a), 7(b), 7(c), and 7(d)). Molecular functions of the identified proteins mainly involved catalytic activity (45.1%), binding (27.5%), transporter activity (9.8%), and structural molecule activity (9.8%). Biological process of the identified proteins mainly involved cellular process (31.2%), metabolic process (23.4%), cellular component organization or biogenesis (11.7%), and localization (10.4%). Cellular component of the identified proteins included cellular part (53.1%), organelles (25%), and macromolecular complex (15.6%). Protein class of the identified proteins mainly involved cytoskeletal protein (17.3%), hydrolase (17.3%), and lyase (9.6%).

### 3.8. Validation of Differentially Expressed Proteins by Western Blot Analysis

**Figure 8:**
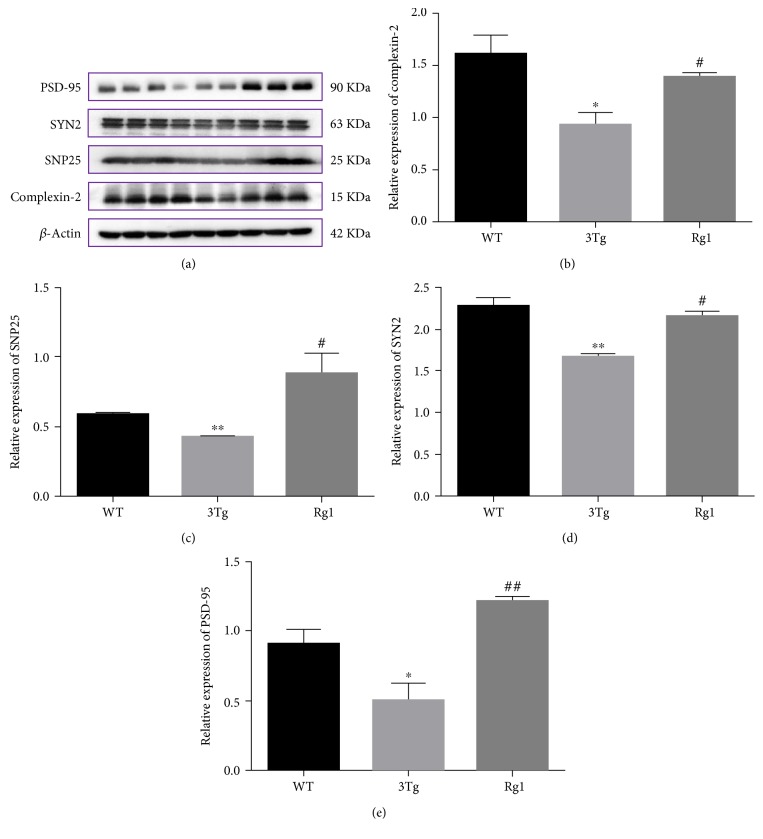
Validation of differentially expressed proteins of CPLX2, SNP25, and SYN2 by Western blot analysis. (a and b) The relative levels of CPLX2 in the hippocampus in WT mice, nontreated 3xTg-AD mice, and Rg1-treated 3xTg-AD mice. (a and c) The relative levels of SNP25 in the hippocampus in WT mice, nontreated 3xTg-AD mice, and Rg1-treated 3xTg-AD mice. (a and d) The relative levels of SYN2 in the hippocampus in WT mice, nontreated 3xTg-AD mice, and Rg1-treated 3xTg-AD mice. (a and e) The relative levels of PSD-95 in the hippocampus in WT mice, nontreated 3xTg-AD mice, and Rg1-treated 3xTg-AD mice. The data were presented as mean ± SEM. ^∗^*p* < 0.05 and ^∗∗^*p* < 0.01 versus WT; ^#^*p* < 0.05 and ^##^*p* < 0.01 versus 3Tg. *n* = 3 for each group.

To confirm the data obtained by 2D-DIGE, Western blot analysis was performed. Three synaptic proteins, CPLX2, SYN2, and SNP25, were selected for further validation. As depicted in Figure 8, in accordance with the results from 2D-DIGE, the expression of CPLX2, SYN2, and SNP25 was significantly downregulated in 3xTg-AD mice relative to the WT mice, while the expression of these proteins was modulated by the treatment of Rg1. Besides, we also determine the expression of PSD-95 in the hippocampus of 3xTg-AD mice. The data showed that the expression of PSD-95 was significantly decreased in 3xTg-AD mice compared with the WT mice (*p* < 0.05), while the expression of this protein was significantly increased after the treatment of Rg1 (*p* < 0.01).

## 4. Discussion

In the present study, we first employed a proteomic approach to investigate the protective effects of Rg1 in 3xTg-AD mice. The 3xTg-AD mice exhibited neurodegenerative changes associated with A*β* and tau deposition that were similar to those observed in patients with AD [28]. Thus, the 3xTg-AD mouse model is a reliable animal model for studies of AD prevention and treatment. Besides, 3xTg-AD mice also displayed obviously synaptic deficit upregulating ryanodine receptor activity which was associated with the symptoms of depression [29]. Overall, 3xTg-AD mice could show the symptom of memory deficit and depression-like behavior.

Additionally, several studies have shown that Rg1 could inhibit morphine-induced spatial memory deficit in both freely moving and anaesthetized rats [30] and produced antidepressant effects via activation of the BDNF signaling pathway upregulating neurogenesis in the hippocampus of mice [31]. Importantly, BDNF, a neurotrophin, plays important roles in protecting and regulating the structure and function of neurons throughout life [32]. Recent studies reported that ginsenoside Rg1 ameliorated hippocampal long-term potentiation and promoted memory [33, 34]. Moreover, Shi et al. also found that Rg1 improved the spatial learning and memory via promoting nonamyloidgenic cleavage of APP, which activated estrogen receptor signaling to MAPK/ERK and PI3K/Akt [35]. Besides, Yang et al. found that long-term Rg1 supplementation improved age-related cognitive decline by promoting synaptic plasticity-associated protein expression, including synaptophysin, N-methyl-D-aspartate receptor subunit 1, postsynaptic density-95, and calcium/calmodulin-dependent protein kinase II alpha [36]. Consistently, we also found that Rg1 upregulated the expression of synaptic proteins such as SYN1, PSD-95, and SNP25 in the hippocampus of 3xTg-AD mice. Therefore, our data demonstrated that the memory impairment and depression-like behavior were ameliorated by the treatment of Rg1, suggesting the protective effects of Rg1 against AD. In addition, we successfully identified 28 proteins in the hippocampus that were differentially expressed in 3xTg-AD mice with or without Rg1 treatment by proteomic analysis.

The behavioral tests, that is, the open field test, the elevated plus maze test, the tail suspension test, and the Morris water maze test, were used to evaluate the anxiety and depression-like behaviors and memory impairment in 3xTg-AD mice. Namely, the open field test and the elevated plus maze were used to explore the anxiety behavior in 3xTg-AD mice, the tail suspension test was used to explore the depression-like behavior in 3xTg-AD mice, and Morris water maze test was used to determine the memory impairment in 3xTg-AD mice. Our data demonstrated that Rg1 treatment could improve depression-like behavior and memory impairment of the 3xTg-AD mice, but its effect on anxiety-like behavior was not obvious, which were consistent with the previous data showing that Rg1 treatment did not produce significant effects on anxiety-like behavior in APP/V7171 transgenic mice bearing the “London” mutant of APP [37, 38], which displayed obvious cognitive impairment and neuroinflammation associated with pathological features of AD. Furthermore, Rg1 was shown to repair hippocampal long-term potentiation (LTP) and improved memory impairment in APP/PS1 mice, likely through facilitating the clearance of AD-associated proteins and activating the BDNF-TrkB pathway [39]. In addition, Rg1 exhibited anti-depressant-like effects in multiple depression-like models [40, 41]. These data collectively demonstrated that Rg1 exerted the neuroprotective effects against memory impairment and depression-like behavior in AD.

Here, the 2D-DIGE technology by using a mixed sample internal standard was applied to identify proteins with distinct molecular functions which could provide a better insight into the underlying molecular mechanisms involved in the neuroprotection of AD of Rg1 treatment. In our study, the proteomic analysis showed that Rg1 treatment altered the expression of hippocampal proteins in 3xTg-AD mice. According to the molecular functions and biological processes, these differentially expressed proteins were mainly involved in synaptic transmission, glycolytic process, response to stress, and regulation of apoptotic process. In the following sections, we focused on the discussion of those differentially expressed proteins associated with memory and depression.

### 4.1. Differentially Expressed Proteins in 3xTg-AD Mice Compared to WT Mice

SYN2, a synaptic vesicle protein, played an important role in neurotransmitter release [42]. The expression of SYN2 was significantly decreased in AD patients [43]. Besides, several studies showed that SYN2 knockout mice displayed obvious cognitive impairment. In combination with our data that the expression of SYN2 was significantly downregulated in 3xTg-AD mice compared with the WT mice, these data indicated an involvement of SYN2 in the regulation of brain functions [44–46]. Though the SYN2 protein was not screened in 3xTg-AD mice with or without Rg1 treatment, the results of Western blot showed that Rg1 treatment significantly altered the expression of SYN2 in 3xTg-AD mice. Thus, it is suggested that Rg1 may exert the protective effects against memory impairment via upregulating the expression of SYN2 in the hippocampus of 3xTg-AD mice.

### 4.2. Differentially Expressed Proteins in 3xTg-AD Mice with or without Rg1 Treatment

SNP25, an important marker of functional synapses, was one of the major proteins involved in the formation of neural soluble N-ethylmaleimide-sensitive factor attachment protein receptor (SNARE) complex [47]. The SNARE complex had a pivotal effect on the central nervous system and was also essential for learning and memory formation [48]. Studies showed that the expression of SNP25 was significantly downregulated in AD brains [49, 50]. In addition, SNP25 expression change was also observed in other forms of dementia, such as vascular and frontotemporal dementia [51, 52]. SNP25 played a vital role in spatial memory ability via regulating glutamate-dependent excitatory transmission [53, 54]. In this study, we found that the expression of SNP25 was significantly decreased in the hippocampus of 3xTg-AD mice compared with the WT mice, while Rg1 treatment could significantly modulate the expression of this protein and alleviate memory impairment of 3xTg-AD mice, suggesting an involvement of SNP25 in memory impairment of 3xTg-AD mice as observed and the protective effects of Rg1 on memory.

CPLX2 was a protein modulator of neurotransmitter release that was downregulated in patients suffering from depression [55]. As a cytosolic protein, a significant change of CPLX2 expression was observed in multiple neurological disorders such as depression [56, 57]. Besides, CPLX-2 knockout mice displayed cognitive disorder [58] and selective impairment in long-term potentiation (LTP) [59]. In our study, we found that the expression of CPLX2 was significantly decreased in 3xTg-AD mice compared with the WT mice and modulated by the treatment of Rg1, suggesting an involvement of CPLX2 in behavioral impairment of 3xTg-AD mice and the protective effects of Rg1 on memory and depression.

### 4.3. Summary

**Figure 9:**
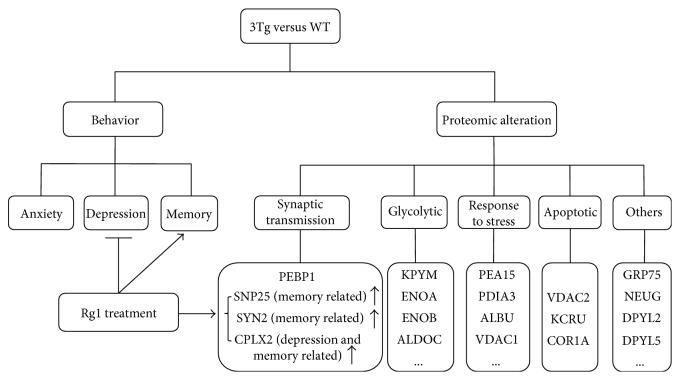
Schematic diagram demonstrating the effects of Rg1 treatment on behavior and hippocampal proteome. On the one hand, Rg1 treatment could improve the memory impairment and ameliorate the depression-like behaviors of 3xTg-AD mice. On the other hand, proteomic analysis revealed that Rg1 could modulate the expression of multiple hippocampal proteins in 3xTg-AD mice. Notably, complexin-2 (CPLX2), a depression- and memory-related protein, and synapsin-2 (SYN2) and synaptosomal-associated protein 25 (SNP25), two memory-related proteins, were significantly downregulated in the hippocampus of 3xTg-AD mice compared with the WT mice, and the treatment of Rg1 modulated the expression of CPLX2 and SNP25 in hippocampus of 3xTg-AD mice.

As displayed in Figure 9, our current data demonstrated that Rg1 could improve memory impairment and depression-like behavior of 3xTg-AD mice and modulate the expression of multiple proteins in the hippocampus. According to the biological function, these differentially expressed proteins were classified into the following 5 categories: synaptic transmission-related proteins, glycolytic process-related proteins, response to stress-related proteins, apoptotic process-related proteins, and other proteins. Importantly, CPLX2, a depression- and memory-related protein, and SYN2 and SNP25, two memory-related proteins, were significantly downregulated in the hippocampus of 3xTg-AD mice, while the expression of these proteins was significantly modulated by the treatment of Rg1 treatment. These data suggested that Rg1 could be used as a potential candidate drug to improve the behavioral deficits in AD via modulating the expression of the proteins (i.e., CPLX2, SYN2, and SNP25) involved in memory and depression behaviors.

## Figures and Tables

**Table 1:** Differentially expressed hippocampus protein spots identified by 2D-DIGE/MALDI-TOF-MS/MS between the WT mice and nontreated 3xTg-AD mice.

Spot number^a^	Accession number	Protein name^b^	MW (Da)^c^	Mascot score	3Tg versus WT	
					*p* value	Ratio^d^
*Modulation of synaptic transmission*						
5	PEBP1_MOUSE	Phosphatidylethanolamine-binding protein 1	20988	198	0.0018	1.11
15	SNP25_MOUSE	Synaptosomal-associated protein 25	23528	393	0.0074	−1.22
29	SYN2_MOUSE	Synapsin-2	63618	128	0.023	−1.19
41	CPLX2_MOUSE	Complexin-2	15499	112	0.034	−1.5
*Glycolytic process*						
2	TPIS_MOUSE	Triosephosphate isomerase	32684	377	0.00026	−1.38
8	PGAM1_MOUSE	Phosphoglycerate mutase 1	28928	546	0.0033	−1.23
33	KPYM_MOUSE	Pyruvate kinase isozymes M1/M2	58378	52	0.027	1.47
34	ENOA_MOUSE	Alpha-enolase	47453	349	0.028	1.21
40	ALDOC_MOUSE	Fructose-bisphosphate aldolase C	39769	350	0.033	−1.08
49	ENOB_MOUSE	Beta-enolase	47337	256	0.048	1.21
50	PGK1_MOUSE	Phosphoglycerate kinase 1	44921	134	0.048	1.27
*Response to stress*						
1	PEA15_MOUSE	Astrocytic phosphoprotein PEA-15	15102	53	0.00024	1.35
10	PDIA3_MOUSE	Protein disulfide-isomerase A3	57099	241	0.0045	1.17
18	ALBU_MOUSE	Serum albumin	70700	676	0.0086	1.24
19	VDAC1_MOUSE	Voltage-dependent anion-selective channel protein 1	32502	99	0.0089	1.11
32	TRFE_MOUSE	Serotransferrin	78841	149	0.027	1.28
45	SODC_MOUSE	Superoxide dismutase [Cu-Zn]	16104	298	0.037	1.11
52	ACTB_MOUSE	Actin, cytoplasmic 1	42052	362	0.049	−1.11
90	ANXA7_MOUSE	Annexin A7	50178	150	0.049	1.14
*Negative regulation of apoptotic process*						
12	VDAC2_MOUSE	Voltage-dependent anion-selective channel protein 2	32340	169	0.0052	1.11
43	KCRU_MOUSE	Creatine kinase U-type, mitochondrial	47373	207	0.036	−1.2
51	COR1A_MOUSE	Coronin-1A	51641	88	0.049	−1.08
*Others*						
3	HBA_MOUSE	Hemoglobin subunit alpha	15133	60	0.0014	−1.26
4	DPYL2_MOUSE	Dihydropyrimidinase-related protein 2	62638	441	0.0016	−1.4
6	SERA_MOUSE	D-3-Phosphoglycerate dehydrogenase	57347	115	0.002	−1.33
7	EF1A1_MOUSE	Elongation factor 1-alpha 1	50424	124	0.0021	1.28
9	DPYL5_MOUSE	Dihydropyrimidinase-related protein 5	62047	141	0.0039	1.23
11	TKT_MOUSE	Transketolase OS = *Mus musculus*	68272	92	0.0048	−1.22
13	GRP75_MOUSE	Stress-70 protein, mitochondrial	73701	244	0.0067	−1.29
16	WDR1_MOUSE	WD repeat-containing protein 1	67049	339	0.0078	−1.4
17	NEUG_MOUSE	Neurogranin	7720	66	0.0082	1.27
21	VATB2_MOUSE	V-type proton ATPase subunit B, brain isoform	56857	337	0.011	1.55
22	HSP7C_MOUSE	Heat shock cognate 71 kDa protein	71055	408	0.012	−1.14
24	B0R1E3_MOUSE	Histidine triad nucleotide-binding protein 1	13601	144	0.013	1.81
25	ATPA_MOUSE	ATP synthase subunit alpha, mitochondrial	59830	279	0.015	−1.14
27	HINT1_MOUSE	Histidine triad nucleotide-binding protein 1	13882	108	0.021	1.21
28	ATPD_MOUSE	ATP synthase subunit delta, mitochondrial	17589	485	0.022	1.37
30	SEP11_MOUSE	Septin-11	50005	66	0.023	1.1
35	PA1B2_MOUSE	Platelet-activating factor acetylhydrolase IB subunit beta	25736	61	0.028	1.15
36	TBA1A_MOUSE	Tubulin alpha-1A chain	50788	103	0.029	−1.2
37	FUMH_MOUSE	Fumarate hydratase, mitochondrial	54550	66	0.031	−1.14
39	ACO13_MOUSE	Acyl-coenzyme A thioesterase 13	15287	97	0.031	1.11
44	F2Z471_MOUSE	Voltage-dependent anion-selective channel protein 1	28254	216	0.036	1.16
47	GT2D1_MOUSE	General transcription factor II-I repeat domain-containing protein 1	124546	35	0.046	1.14
48	CALM_MOUSE	Calmodulin	16827	338	0.046	1.33
53	PSA2_MOUSE	Proteasome subunit alpha type-2	26024	190	0.049	1.19
67	DHSA_MOUSE	Succinate dehydrogenase [ubiquinone] flavoprotein subunit, mitochondrial	73623	192	0.025	−1.33

^a^Protein ID assigned manually. ^b^Protein name identified by MALDI-TOF-MS/MS. ^c^Theoretical molecular weight of the proteins. ^d^The ratio in spot intensity from 3xTg-AD mice compared to WT mice. *N* = 6 for each group.

**Table 2:** Differentially expressed hippocampus protein spots identified by 2D-DIGE/MALDI-TOF-MS/MS between 3xTg-AD mice treated with and without Rg1.

Spot number^a^	Accession number	Protein name^b^	MW (Da)^c^	Mascot score	Rg1-3Tg versus 3Tg	
					*p* value	Ratio^d^
*Modulation of synaptic transmission*						
15	SNP25_MOUSE	Synaptosomal-associated protein 25	23528	393	0.045	1.17
41	CPLX2_MOUSE	Complexin-2	15499	112	0.0016	1.66
58	SYN1_MOUSE	Synapsin-1	74223	96	0.012	−1.39
63	NSF-MOUSE	Vesicle-fusing ATPase	83131	63	0.019	−1.26
*Glycolytic process*						
14	PGAM1_MOUSE	Phosphoglycerate mutase 1	28928	623	0.0067	1.32
33	KPYM_MOUSE	Pyruvate kinase isozymes M1/M2	58378	52	0.036	−1.12
69	ENOA_MOUSE	Alpha-enolase	47453	136	0.0092	−1.12
*Response to stress*						
19	VDAC1_MOUSE	Voltage-dependent anion-selective channel protein 1	32502	99	0.047	−1.08
32	TRFE_MOUSE	Serotransferrin	78841	149	0.0021	−1.39
61	1433Z_MOUSE	14-3-3 protein zeta/delta	27925	151	0.01	1.19
72	CAH2_MOUSE	Carbonic anhydrase 2	29129	195	0.018	1.14
74	GLNA_MOUSE	Glutamine synthetase	42834	412	0.023	1.09
90	ANXA7_MOUSE	Annexin A7	50178	150	0.0072	−1.22
*Negative regulation of apoptotic process*						
2	TPIS_MOUSE	Triosephosphate isomerase	32684	377	6.60*E*−06	1.31
18	ALBU_MOUSE	Serum albumin	70700	676	0.023	−1.24
77	GSTP1_MOUSE	Glutathione S-transferase P 1	23765	434	0.04	1.14
*Others*						
4	DPYL2_MOUSE	Dihydropyrimidinase-related protein 2	62638	441	0.031	1.29
6	SERA_MOUSE	D-3-Phosphoglycerate dehydrogenase	57347	115	0.016	1.21
7	EF1A1_MOUSE	Elongation factor 1-alpha 1	50424	124	0.043	−1.16
24	B0R1E3_MOUSE	Histidine triad nucleotide-binding protein 1	13601	144	0.003	−1.74
56	B1AXW5_MOUSE	Peroxiredoxin-1 (fragment)	19086	379	0.00015	1.21
68	TMOD2_MOUSE	Tropomodulin-2	39487	81	0.0052	1.1
70	DHPR_MOUSE	Dihydropteridine reductase	25782	360	0.0094	1.1
73	E0CZA1_MOUSE	T-complex protein 1 subunit epsilon (fragment)	21569	49	0.019	−1.32
75	G5E8R0_MOUSE	Tropomyosin 1, alpha, isoform CRA_i	28383	275	0.034	1.15
76	GBB2_MOUSE	Guanine nucleotide-binding protein G(I)/G(S)/G(T) subunit beta-2	38048	428	0.039	1.05
78	VATB2_MOUSE	V-type proton ATPase subunit B, brain isoform	56857	350	0.042	−1.93
79	ACTG_MOUSE	Actin, cytoplasmic 2	42108	176	0.049	1.08

^a^Protein ID assigned manually. ^b^Protein name identified by MALDI-TOF-MS/MS. ^c^Theoretical molecular weight of the protein(s). ^d^The ratio in spot intensity from Rg1-treated 3xTg-AD mice compared to nontreated 3xTg-AD mice. *N*=6 for each group.
